# Thyroid dysfunction in young, first-episode and drug-naïve patients with major depressive disorder: prevalence and associated clinical factors

**DOI:** 10.3389/fpsyt.2023.1156481

**Published:** 2023-06-30

**Authors:** Jinbo Wu, Zhunian Wang, Hongjiao Xu, Liying Yang, Jiacheng Liu, Yue Zheng, Chuanyi Kang, Xiaohong Wang, Jingjing Shi, Na Zhao, Xiang Yang Zhang

**Affiliations:** ^1^First Affiliated Hospital of Harbin Medical University, Harbin, Heilongjiang Province, China; ^2^Qingdao Mental Health Center, Qingdao, China; ^3^Dalian No.7 People's Hospital, Dalian, Liaoning Province, China; ^4^Peking University Sixth Hospital, Beijing, China; ^5^CAS Key Laboratory of Mental Health, Institute of Psychology, Chinese Academy of Sciences, Beijing, China

**Keywords:** major depressive disorder, prevalence, association, metabolize, thyroid dysfunction

## Abstract

**Objective:**

The incidence of thyroid dysfunction (TD) and major depressive disorder (MDD) is increasing year by year in the general population. However, the prevalence and correlates of TD in first-episode drug-naive (FEDN) MDD patients have not been explored. This study sought to fill this gap and examine the association between TD and MDD.

**Methods:**

We recruited 1,289 FEDN MDD patients aged 18 ~ 45 years. A total of 1,289 FEDN MDD outpatients were recruited. Demographical and suicide data were collected for each patient, and lipid profiles, thyroid function, and fasting blood glucose (FBG) levels were measured. The Hamilton Depression Scale 17 (HAMD-17) was assessed for depression.

**Results:**

The prevalence of TD in young FEDN MDD patients was 64.86%. Compared with those without TD, patients with TD had longer duration of illness, greater HAMD score, higher BMI, TG, TC, and LDL-C levels, and higher suicide attempt rates, but lower HDL-C and FBG levels. Further logistic regression indicated that duration of illness, HAMD score, TC, HDL-C, BMI, and FBG levels were significantly associated with TD.

**Limitations:**

No causal relationship can be drawn due to the cross-sectional design.

**Conclusion:**

TD is common in young FEDN MDD patients. So clinicians should monitor thyroid function in patients with MDD.

## 1. Introduction

Thyroid dysfunction (TD) is associated with various psychiatric disorders, such as depression (1), mania (2, 3), acute psychosis (4, 5), insomnia (6), and cognitive dysfunction (7). TD was defined as abnormal thyroid function test results (8) and includes overt hyperthyroidism, overt hypothyroidism, subclinical hypothyroidism, subclinical hyperthyroidism, and normal thyroid pathology syndrome (9). The diagnosis of thyroid disease is based on evidence of structural abnormalities and altered gland secretory function. The hormones secreted by the thyroid gland control most tissues’ functions and maintain the body’s internal homeostasis. In a study of TD in the Chinese population, it was found that TD affect the metabolism of both male and female in different ways (10), including metabolism, cell differentiation, and neurodevelopment, male and female show sex differences in neuronal signaling. Steroid hormones, including testosterone and estrogen, are thought to be the primary regulators of neuronal signaling programmed in a male- and female-specific manner (11). A study has shown that thyroid hormone, acts primarily through its nuclear receptors and that deficiency of this hormone may lead to mild (mood disorders in adulthood) to severe neurological impairment; T3 is responsible for the expression of numerous central nervous system genes related to oxygen transport, growth factors, myelination, and cell maturation (12). The prevalence of TD in the general population varies widely, ranging from 6.6 to 13.4% (13, 14). The data suggest that the number of people with TD in China may have exceeded 200 million; however, the awareness rate of patients with thyroid disease in China is only 18% (15).

Major depressive disorder (MDD) is thought to be accompanied by mild neuroendocrine disorders, including thyroid disease. Only a small proportion of depressed patients have been found to have significant thyroid disease, but an association between changes in the Hypothalamus-Pituitary-Testis (HPT) Axis and depression has been demonstrated (16). In a study on the pathology of depression, thyroid hormone levels in hair varied with episodes of depression (17). A previous study noted that refractory depression is often associated with subclinical hypothyroidism (18). Notably, the symptoms of TD are often insidious and share many similarities with the manifestations of suboptimal health in today’s fast-paced lifestyles. Most people attribute symptoms such as weight changes, irritability, anxiety, insomnia, and overwork to psychological stress and lifestyle, without realizing that TD is the underlying cause. Missing a TD diagnosis may lead not only to depression or mood cycling, but also to a delayed response to treatment. The question is whether MDD is the cause of TD and vice versa. However, previous studies of TD in patients with MDD have had mixed results due to various factors, including heterogeneity of depression, antidepressant medication, age of onset, and even inpatient/discharge patient status, which may have contributed to these inconsistent results.”

There are inconsistent findings regarding the effect of age on thyroid hormones. Some domestic studies exploring specific reference intervals for normal thyroid hormone testing in the Chinese population remain controversial (19, 20). Some studies have found lower T4 and T3 levels in female aged 16 to 49 years than in male in the same age group, but no such differences were found in older subjects (21). Not surprisingly, thyroid hormones can affect cognitive levels. Even patients with mild thyroxine dysfunction may have mental deficits or psychomotor disorders (22, 23). Changes in reproductive hormone levels during menstruation, pregnancy, postpartum, perimenopause, and menopause may influence susceptibility to mood disorders, as these hormones may regulate neurohormonal, neurotransmitter, and biological clock mechanisms (24). In patients with mood disorders, the additional burden of thyroid dysfunction may have significant consequences, affecting the psychiatric symptoms presented, treatment choices and responses, and clinical monitoring requirements, but the mechanisms are unclear; therefore, it is crucial to explore the tools of age differences in thyroid dysfunction in depressed patients.

The great potential of selective thyroid hormones in targeting various human pathologies characterized by changes in metabolism or cellular differentiation has been demonstrated (25). Alterations in hormonal and endocrine function may influence the pathophysiological mechanisms of depression. Thyroid hormones are crucial in affecting neural stem cells in the adult brain (26). Glial cells regulate immune responses, modulate neurotransmitters’ release, and control neurons’ metabolism. The interaction between thyroid hormones and glial cells is also relevant to the adult brain (27); it may be a novel target for improving cognitive function in young first-episode drug-naive (FEDN) depressed patients (28). It may be an intervention for disease onset and progression (29, 30). While the increased incidence of thyroid disease and MDD in younger people makes it necessary for us to explore the incidence in younger populations. Therefore, the present study examined the prevalence and correlates of TD in young Chinese patients with FEDN MDD aged 18 to 45 (31–33).

## 2. Methods

### 2.1. Subjects

All subjects were recruited from 2015 to 2017 at the psychiatric outpatient clinic of a general hospital in Taiyuan, Shanxi Province, China. After a detailed explanation, all enrolled subjects were required to sign an informed consent form. The work described was performed in accordance with the ethical guidelines of the World Medical Association as specified below (Declaration of Helsinki). The ethics committee approved the implementation of this study at the First Affiliated Hospital of Shanxi Medical University (No. 2016-Y27).

In this cross-sectional study, a total of 1,289 patients who met the study criteria were finally recruited. All participants met the following inclusion criteria: (1) age between 18 and 45 years; (2) meeting the diagnosis of MDD in the Diagnostic and Statistical Manual of Mental Disorders, Fourth Edition (DSM-IV) by two trained psychiatrists; (3) 17-item Hamilton Depression Rating Scale (HAMD-17) ≥24; (4) depressive symptoms as a first-episode with a disease duration of no more than 24 months; (5) no previous treatment with antidepressants, antipsychotics, and any other medications before.

Exclusion criteria were: (1) serious physical illness; (2) received thyroxine treatment or treatment for thyroid dysfunction; (3) other serious mental illness; (4) history of drug or alcohol abuse except for nicotine; (5) pregnant or breastfeeding women; and (6) refusal to sign an informed consent form.”

### 2.2. Socio-demographic information and clinical measures

Our study used a questionnaire to collect basic demographic information and clinical data from all subjects, including age (years), gender (male, female), duration of illness (months), age of onset (years), suicide attempt (yes, no), marital status (married or unmarried), education (middle school, high school, undergraduate, graduate) and body mass index (BMI).

The 17-item HAMD (HAMD-17) was used to assess the severity of depressive symptoms (34), with eight items scoring from 0 to 4 and nine items scoring from 0 to 2. Two research psychiatrists were trained to measure the scale before the study started. After repeated assessments, the interobserver correlation coefficient for the HAMD total score exceeded 0.8.

### 2.3. Measurement of biochemical parameters and thyroid function

Biomarker samples from each participant were collected between 6 and 8 a.m. to determine blood measurements, then centrifuged and stored at −20 degrees for testing. An electrochemiluminescent immunoassay was used to determine the concentration of thyroid function in the serum. An enzymatic colorimetric assay was used to detect lipid profiles.

Biochemical parameters measured in our study included total cholesterol (TC), triglycerides (TG), high-density lipoprotein cholesterol (HDL-C), and low-density lipoprotein cholesterol (LDL-C), as well as thyroid function-related parameters, including thyrotropin-releasing hormone (TSH), free triiodothyronine (FT3), free thyroxine (FT4), anti-thyroid peroxidase antibodies (A-TPO) and anti-thyroglobulin antibodies (A-TG). In addition, we measured fasting blood glucose (FBG) levels.

We divided all enrolled patients into two groups: with or without TD. Based on previous studies in Chinese populations, the definition of thyroid dysfunction is explained as follows: (1) abnormal TgAb: TgAb ≥115 IU/L; (2) abnormal TPOAb: TPOAb ≥34 IU/L; (3) subclinical hypothyroidism (SCH): TSH > 4.2 mIU/L and normal fT4 concentration(10–23 pmol/L); (4) hyperthyroidism: TSH < 0.27 mIU/L, FT4 > 23 pmol/L; (5) hypothyroidism: TSH > 4.2 mIU/L and low FT4 concentration (<10 pmol/L).

### 2.4. Statistical analysis

The data were analyzed using SPSS 23.0 statistical software. All variables were described as mean ± standard deviation or percentage. After grouping, a one-sample Kolmogorov–Smirnov (K-S) test was performed to test for normal distribution. ANOVA was performed to compare measurements that conformed to a normal distribution between the two groups, and nonparametric tests were performed to compare variables that were not normally distributed. ANOVA was used to compare demographic and clinical variables in the group with and without TD, and chi-square tests were performed for categorical variables. A binary logistic regression analysis was then performed with the presence or absence of TD as the dependent variable. After univariate analysis, socio-demographic and clinical data with significant differences were used as covariates to analyze independent factors affecting TD in young FEDN MDD patients. A significant value of p of 0.05 (two-tailed) was set for our study. Bonferroni correction was adopted to adjust for multiple tests.

## 3. Results

### 3.1. Demographic and characteristics of patients

A total of 1,289 patients who met the inclusion and exclusion criteria were included in our study, including 470 male and 819 famle. The mean age of the patients was 29.29 years [standard deviation (SD) = 8.68], ranging from 18 to 45 years. The mean duration of the disease was 5.65 months (SD = 4.10), the mean age of onset was 29.13 years (SD = 8.59), and the mean BMI level was 24.35 Kg/m^2^ (SD = 1.95).

Two hundred and forty-four (18.9%) patients had attempted suicide, and 802 (62.2%) were married. In addition, 181 (14.0%) were junior high school students, 611 (47.4%) were high school students, 411 (31.9%) were undergraduate students, and 86 (6.7%) had postgraduate degrees.

### 3.2. Demographic and clinical characteristics of young FEDN MDD patients with and without TD

The demographic and clinical characteristics of young FEDN MDD patients are shown in Table 1. The prevalence of TD among young Chinese FEDN patients was 64.86% (836/1289). Compared to the non-TD group, the TD group had a longer duration of illness (*F* = 16.274, *p* < 0.001), higher HAMD score (*F* = 202.888, *p* < 0.001), FBG (*F* = 187.610, *p* < 0. 001), TC (*F* = 226.741, *p* < 0.001), TG (*F* = 7.619, *p* = 0.006), lower HDL-C (*F* = 68.893. *p* < 0.001), LDL-C (*F* = 113.432, *p* < 0.001) and BMI levels (*F* = 22.595, *p* < 0.001) and a higher rate of suicide attempts (2 = 38.660, *p* < 0.001). They all passed the Bonferroni correction (*p* < 0.05/13 = 0.0038).

**Table 1:** Demographic and clinical characteristics of young FEDN MDD patients with and without TD (*n* = 1,289).

	All patients	without TD	with TD	F/χ2	*p*
Sample size	1,289	453	836		
Age	29.29 (8.68)	28.65 (8.62)	29.63 (8.70)	3.747	0.054
Duration of illness	5.65 (4.10)	5.03 (4.07)	5.99 (4.075)	16.274	<0.001
Age of onset	29.13 (8.59)	28.51 (8.54)	29.47 (8.60)	3.627	0.057
HAMD	30.18 (2.92)	28.72 (2.54)	30.97 (2.80)	202.888	<0.001
FBG	5.375 ± 0.637	5.541 ± 0.630	5.066 ± 0.527	187.610	<0.001
TC	5.20 (1.10)	4.62 (0.88)	5.51 (1.08)	226.741	<0.001
TG	2.17 (1.00)	2.07 (1.03)	2.23 (0.97)	7.619	0.006
HDL-C	1.23 (0.28)	1.32 (0.23)	1.18 (0.30)	68.893	<0.001
LDL-C	2.94 (0.86)	2.61 (0.73)	3.12 (0.88)	113.432	<0.001
BMI	24.35 (1.95)	24.00 (1.78)	24.54 (2.01)	22.595	<0.001
Suicide attempts				38.660	<0.001
Yes	244 (18.9%)	44 (9.7%)	200 (23.9%)		
No	1,045 (81.1%)	409 (90.3%)	636 (76.1%)		
Marital status				2.391	0.122
Yes	802 (62.2%)	175 (59.4%)	533 (63.8%)		
No	487 (37.8%)	184 (40.6%)	303 (36.2%)		
Education level				0.107	0.991
Junior high school	181 (14.0%)	64 (14.1%)	117 (14.0%)		
High school	611 (47.4%)	217 (47.9%)	394 (47.1%)		
Undergraduate	411 (31.9%)	142 (31.3%)	269 (32.2%)		
Postgraduate	86 (6.7%)	30 (6.6%)	56 (6.7%)		

BMI, body mass index; FBG, fasting blood glucose; FEDN, first-episode and drug naïve; HAMD, Hamilton Rating Scale; HDL-C, high-density lipoprotein cholesterol; LDL-C, low-density lipoprotein cholesterol; MDD, major depressive disorder; TD, thyroid dysfunction; TC, total cholesterol; TG, triglycerides.

### 3.3. Results of binary logistic regression analysis: associated factors for TD in young FEDN MDD patients

Stepwise logistic regression analysis was performed to identify associated factors for TD. As shown in Table 2, the independent associated factors for TD in young FEDN MDD patients were duration of illness (OR = 0.021, 95% CI = 1.006–1.078, *p* = 0.021), HAMD total score (OR = 1.173, 95% CI = 1.104–1. 241, *p* < 0.001), TC (OR = 1.225, 95% CI = 1.111–1.351, *p* < 0.001), HDL-C (OR = 0.232, 95% CI = 0.137–0.396, *p* < 0.001), BMI (OR = 1.142, 95% CI = 1.062–1.229, *p* < 0.001) and FBG levels (OR = 2.999, 95% CI = 2.318–3.880, *p* < 0.001).

**Table 2:** Demographic and clinical variables independently associated with TD.

Variables	B	Wald statistic	*p*	OR	95% CI
Duration of illness	0.041	5.363	0.021	1.042	1.006–1.078
HAMD	0.160	26.394	<0.001	1.173	1.104–1.247
TC	0.441	20.180	<0.001	1.555	1.283–1.885
HDL-C	−1.459	28.857	<0.001	0.232	0.137–0.396
BMI	0.133	12.682	<0.001	1.142	1.062–1.229
FBG	1.098	69.844	<0.001	2.999	2.318–3.880

BMI, body mass index; FBG, fasting blood glucose, HAMD, Hamilton Rating Scale; HDL-C, high-density lipoprotein cholesterol; TC, total cholesterol; TD, thyroid dysfunction.

## 4. Discussion

This is the first study to examine the prevalence of TD and associated factors in young FEDN MDD patients. In this study, the prevalence of TD in young FEDN MDD patients was 64.86%. Further, compared with patients without TD, patients with TD had an abnormal lipid profile, as evidenced by higher TC, TG, and LDL-C levels and lower HDL-C levels. Further logistic regression showed that high TC, TG, and low HDL-C were associated factors in the development of TD. Moreover, long duration of disease, severe depressive symptoms and high FBG levels were also associated factors for the development of TD.

An important finding in our study was that the prevalence of TD in young patients with FEDN MDD was 64.86%, which differs from previous studies. For example, Sintzel et al. reported that the prevalence of subclinical hypothyroidism in depressed patients was 8–17% and up to 52% in patients with treatment-resistant depression (35). In a study of 263 depressed patients, 69 (26.2%) of them had TD, 12.2% had subclinical hypothyroidism, 4.9% had overt hypothyroidism, and 2.7% had overt hyperthyroidism (1). There are several reasons for the inconsistent results. One of the main reasons is the different populations enrolled; in our study, emphasis was placed on patients with first-onset disease and no previous medication use. Although previous studies point to a higher prevalence in older adults (36), the prevalence of thyroid disease and depression in young people is increasing as the rapid economic development nowdays exposes them to a high level of life and financial stress leaving many young people in a state of chronic inflammation. In addition, MDD has been diagnosed to be associated with neurotransmitter abnormalities, which likewise lead to pretend present dysfunction (37), and the prevalence of TD in young MDD patients is also on the rise, stimulated by chronic stressors.

In the present study, we found that the severity of depression was an associated factor for the development of TD. Earlier clinical studies have noted an association between the severity of depression and thyroid function (38, 39). For example, untreated diagnosed hypothyroidism was positively correlated with Beck Depression Inventory (BDI-II) scores (40). It is not difficult to understand the alterations in the hypothalamic–pituitary-thyroid axis (HPT) in patients with primary depression and the dramatic changes in the HPT axis that occur with increasing severity and duration of depression (41). Also, it is not difficult to imagine that more severe depressive symptoms could mean that they have disturbed sleep rhythms, irregular diet, and less physical exercise, which in turn lead to abnormal thyroid function (42). On the other hand, MDD is associated with chronic overactivity of the stress system (43), with increased pro-inflammatory and oxidative stress responses and subsequent hyperactivation of the HPA axis as MDD progresses (44). However, further studies are needed to clarify this finding in the future. Therefore, focusing on MDD patients with a long duration of disease and high degree of depression would certainly facilitate the early detection of TD.

The thyroid gland plays a crucial role in metabolic regulation and TD is associated with metabolic parameters such as FBG and lipid metabolism, blood pressure levels and BMI (45). Previous studies have shown an association between TD and lipids or depression (46, 47). However, few studies have explored the relationship between these three. Similar to our study, health and nutrition screening data from 4,275 Korean nationals showed significant differences in the prevalence of TD (48). Regarding the explanation of the mechanism, some studies suggest that it may be related to cholesterol excretion, changes in lipoprotein B, and the activity of hepatic lipase and cholesteryl ester transport proteins (49–51). This study also found that BMI was higher in patients with TD and logistic analysis also showed that high BMI was a relevant factor in the development of TD. A study on the incidence of depression in patients with TD and central obesity found that serum FT4 levels were negatively correlated with BMI, but TSH levels were positively correlated with BMI (52). Notably, a retrospective observational study showed that many MDD patients were overweight (53). Leptin has been implicated in thyroid function to varying degrees, and the development of resistance to the weight-lowering effects of leptin in obese patients is likely to be initiated by activation of inflammatory signaling, which largely promotes dysregulation of the immune response and propagation of autoimmunity in susceptible individuals (54). The possible mechanism is the increased activity of thyroxine 5-deiodinase, an enzyme that converts T4 to T3 and is a crucial mechanism of thyroid hormone regulation in people with high BMI (55). Adding metabolic screening parameters associated with patients with MDD, especially those who are obese, may help to detect their thyroid problems.

The co-prevalence of depression, thyroid disease, and diabetes is known to be high. Still, it is difficult to determine the relationship between these three, considering the effects of antidepressants on the endocrine system. To exclude the impact of medications and aging on blood glucose and thyroid hormones, the patients included in this study were young patients with a first episode and previously untreated with drugs. Our study found a strong association between FBG levels and TD in young FEDN MDD patients. Hypothyroidism exhibits considerable alterations in blood flow and glucose metabolism in the brain (56). Several studies have found that abnormal blood glucose regulation can lead to TD (57). In an animal experiment, mice that developed TD exhibited hyperglycemia, reduced tissue glycogen content in the liver and skeletal muscle, increased glycogen content in the heart and kidneys, and histological sections of the pancreas showing destruction (58). Abnormal thyroid hormone secretion may affect blood glucose levels by influencing the secretion of insulin-like factor-1, resistin, and insulin (59). Therefore, control of FBG is essential in patients with depression.

Our study still has some limitations worth mentioning. First, these results were obtained from a cross-sectional design survey. Therefore, they cannot be used as valid evidence for a causal relationship between TD and associated risk factors. Our future studies should include longitudinal or retrospective cohort studies. Second, the patients included in this study were from China and were recruited from outpatient clinics. Although the effect of medication on outcome was excluded, it cannot be generalized to all patients with depression. Therefore, the results of our study should be evaluated in other populations from different countries and clinical settings. Third, confounding factors, such as smoking and alcohol consumption, were not included in this study. These factors also play an important role in depression, TD, or dyslipidemia. In the future, more confounding factors will be retained for further exploration, which will help clarify potential mechanisms to establish a more accurate association. Fourth, TD manifests itself in different ways, which is very broad, and in the future we need to refine each symptom so that clinicians can have more precise treatment directions.

In conclusion, this study demonstrated a high prevalence of TD in young FEND MDD patients. Long-term disease duration, higher TC, BMI, FBG, and low HDL-C levels were independently associated factors with TD in young FEND MDD patients. Targeting abnormal TC, FBG, HDL-C and longer duration of illness in patients with MDD may help to reduce thyroid dysfunction in young FEDN patients. Longitudinal studies with large sample sizes are needed to further elucidate the relationship between sex-specific factors and TD in MDD patients.

## Data availability statement

The original contributions presented in the study are included in the article/supplementary material, further inquiries can be directed to the corresponding author.

## Ethics statement

The studies involving human participants were reviewed and approved by The first Affiliated Hospital of Shanxi Medical University. The patients/participants provided their written informed consent to participate in this study.

## Author contributions

JW, NZ, and XZ were responsible for study design, statistical analysis, and manuscript preparation. ZW, HX, LY, JL, YZ, CK, XW, and JS were responsible for recruiting the patients and the data curation. NZ and XZ were involved in evolving the ideas and editing the manuscript. All authors contributed to the article and approved the submitted version.

## Funding

This work was supported by the First Affiliated Hospital of Harbin Medical University (grant number 2021B22) and the Natural Science Foundation of Heilongjiang Province (grant number LH2022H040).

## Conflict of interest

The authors declare that the research was conducted in the absence of any commercial or financial relationships that could be construed as a potential conflict of interest.

## Publisher’s note

All claims expressed in this article are solely those of the authors and do not necessarily represent those of their affiliated organizations, or those of the publisher, the editors and the reviewers. Any product that may be evaluated in this article, or claim that may be made by its manufacturer, is not guaranteed or endorsed by the publisher.
